# Sentinel Node Biopsy for Head and Neck Melanoma: A 12-Year Experience from a Medium-Volume Regional Center

**DOI:** 10.3390/jcm15020763

**Published:** 2026-01-17

**Authors:** Péter Lázár, Kristóf Boa, Noémi Mezőlaki, Zoltán Varga, Zsuzsanna Besenyi, Erika Varga, István Balázs Németh, Eszter Baltás, Judit Oláh, Erika Gabriella Kis, József Piffkó, Róbert Paczona

**Affiliations:** 1 Department of Oral- and Maxillofacial Surgery, University of Szeged, 6720 Szeged, Hungary; boa.kristof@gmail.com (K.B.); office.maxillo@med.u-szeged.hu (J.P.);; 2 Department of Dermatology and Allergology, University of Szeged, 6720 Szeged, Hungary; noemimezolaki@gmail.com (N.M.); varga.erika@med.u-szeged.hu (E.V.); nemethistvanbalazs@gmail.com (I.B.N.); baltas.eszter@med.u-szeged.hu (E.B.); ksgbrll@gmail.com (E.G.K.); 3 Department of Oncotherapy, University of Szeged, 6720 Szeged, Hungary; varga.zoltan@med.u-szeged.hu (Z.V.); office.onko@med.u-szeged.hu (J.O.); 4 Department of Nuclear Medicine, University of Szeged, 6720 Szeged, Hungary; office.numed@med.u-szeged.hu

**Keywords:** sentinel lymph node biopsy, head and neck melanoma, cutaneous melanoma, staging, prognosis

## Abstract

**Background**: Head and neck (H&N) cutaneous melanomas have poorer outcomes than melanomas at other sites, yet sentinel lymph node biopsy (SLNB)—a key prognostic tool in clinically node-negative disease—is less frequently performed, particularly outside tertiary centers. We evaluated the feasibility and prognostic relevance of SLNB in a medium-volume regional institution. **Methods**: We retrospectively reviewed patients with primary H&N cutaneous melanoma who underwent SLNB at the Department of Oral and Maxillofacial Surgery, University of Szeged, between 2010 and 2022. Clinicopathological features, nodal outcomes, recurrence patterns, recurrence-free survival (RFS), and overall survival (OS) were analyzed using Kaplan–Meier methods and univariate Cox regression. **Results**: Thirty-eight patients underwent SLNB, with a 100% sentinel lymph node identification rate and no major complications. Positive sentinel lymph nodes were identified in 8 patients (21.1%). Two false-negative events occurred, resulting in a false-omission rate of 6.7% and a negative predictive value of 93.3%. SLN-negative patients demonstrated longer RFS and OS, although differences were not statistically significant. Among patients with intermediate-risk melanoma (pT1b–pT3a), 18.5% had a positive SLN. **Conclusions**: SLNB is a safe and clinically meaningful staging procedure for H&N melanoma in a medium-volume regional center. Sentinel node status provides important prognostic information and supports appropriate patient selection for contemporary adjuvant therapy.

## 1. Introduction

The incidence of head and neck (H&N) melanoma has been steadily increasing, and approximately one-fifth of all cutaneous melanomas arise in this anatomically complex region [1]. H&N melanomas are associated with a poorer prognosis compared with those arising at other anatomical sites [2,3]. Therefore, precise disease staging and the prompt initiation of individualized, multidisciplinary treatment are particularly important in these cases.

Sentinel lymph node biopsy (SLNB), first described by Morton and Cochran in 1992 [4], is a widely accepted method for identifying lymph node micrometastases. Sentinel lymph node (SLN) status has been recognized as one of the most significant independent prognostic factors in melanoma, and its accuracy, prognostic value, and low morbidity were established in large multicenter randomized trials, most notably the Multicenter Selective Lymphadenectomy Trial I (MSLT-I) [5,6]. In clinically node-negative patients, SLNB enables accurate locoregional pathological staging, thereby improving risk assessment and facilitating timely decisions regarding adjuvant therapy. Current international guidelines recommend performing SLNB for melanomas with a tumor thickness of ≥0.8 mm or <0.8 mm with ulceration (pT1b stage or higher, according to the 8th AJCC TNM classification and NCCN guidelines) [7,8].

In recent years, melanoma management has evolved from a predominantly surgical approach toward a more individualized, systemic treatment paradigm [9,10]. Alongside treatment efficacy, maintaining patient quality of life has become a central consideration. Given the considerable functional and aesthetic morbidity associated with neck dissections, their avoidance has emerged as a key therapeutic objective. Completion lymph node dissection (CLND), once the standard intervention following a positive SLNB, has now been largely replaced by systemic targeted and immunotherapeutic options [11,12,13]. Consequently, the principal role of SLNB has shifted from regional disease control to serving primarily as a staging tool that guides patient selection for systemic adjuvant treatment [14,15,16].

Despite its established prognostic value and therapeutic relevance in H&N cutaneous melanoma, SLNB is not routinely performed in many oncology centers. This variability in clinical practice is also evident in Hungary, where SLNB for H&N melanoma is available only in a limited number of specialized cancer centers. Consequently, a substantial proportion of patients with H&N melanoma may not have access to guideline-recommended, contemporary oncologic care. Recent data indicate that patients with H&N melanomas are significantly less likely to undergo SLNB compared with those with melanomas at other anatomical sites [17]. Similarly, another large population-based assessment demonstrated that SLNB rates were lowest for tumors located on the head and neck [18].

This hesitancy is often attributed to historically higher false-negative rates associated with SLNB in the head and neck region compared with other anatomical sites [19,20]. However, evidence from high-volume H&N centers has increasingly challenged this perception. These centers have demonstrated SLN identification and accuracy rates comparable to those achieved in other anatomical regions, supporting its broader integration into standard clinical practice [21].

An important consideration is whether SLNB can be performed with comparable accuracy and oncologic reliability in medium-volume regional oncology centers. Early reports describing the technique suggested a learning curve of approximately 30–40 cases [22,23]. Nevertheless, accumulating clinical experience indicates that when essential prerequisites are fulfilled—namely, the availability of high-quality nuclear medicine imaging, experienced histopathological assessment, and surgical expertise in head and neck anatomy—the learning curve can be significantly shortened. In this context, a well-coordinated multidisciplinary approach may allow regional institutions to achieve outcomes comparable to those of high-volume tertiary centers.

The objective of the present study was to evaluate the clinical characteristics and long-term oncologic outcomes of patients with H&N cutaneous melanoma who underwent SLNB at the Department of Oral and Maxillofacial Surgery, University of Szeged, a medium-volume regional head and neck surgical center in Hungary. The findings were compared with data reported from previously published series to assess the feasibility, reliability, and oncologic outcomes of performing SLNB in a non-tertiary institutional setting.

## 2. Materials and Methods

### 2.1. Study Design and Setting

This retrospective single-center study included patients with primary cutaneous head and neck melanoma who underwent surgical treatment, including SLNB, between 2010 and 2022 at the Department of Oral and Maxillofacial Surgery, University of Szeged. Demographic and clinicopathological variables—including age, sex, tumor location, Breslow thickness, ulceration, mitotic rate, and the presence of histological regression—were extracted from the institutional electronic medical record system (MEDSOL).

At our institution, the management of cutaneous melanoma is coordinated by the Department of Dermatology, with all cases reviewed at the Multidisciplinary Skin Tumor Board of the Albert Szent-Györgyi Medical Center. Decisions regarding staging procedures, including SLNB, are made during these meetings in accordance with contemporaneous guideline recommendations, tumor-related risk factors, and individual patient considerations.

All SLNB procedures included in the present analysis were performed at the Department of Oral and Maxillofacial Surgery by surgeons specialized in head and neck oncologic surgery, following standardized institutional protocols. Restricting the analysis to procedures performed within this department ensured consistency in surgical technique, perioperative management, histopathological evaluation, and follow-up.

### 2.2. Patient Selection and Eligibility

All consecutive patients with primary cutaneous head and neck melanoma who underwent surgical treatment, including SLNB, at the Department of Oral and Maxillofacial Surgery during the study period were retrospectively identified. Sentinel lymph node biopsy was performed in a selected subgroup of patients based on contemporaneous guideline recommendations, multidisciplinary tumor board decision-making, tumor-related risk factors, and the anticipated impact of SLNB findings on subsequent management.

Indications for SLNB evolved over the 12-year study period. During the earlier years of the study, SLNB was generally recommended for patients with clinically node-negative disease and a Breslow thickness ≥1.0 mm, in accordance with prevailing international guidelines at that time. Following subsequent guideline updates, the threshold was lowered to ≥0.8 mm, and additional high-risk histopathological features were considered. For the purposes of analysis, all tumors were reclassified according to current AJCC criteria to ensure consistency across the cohort. No pT1a melanomas underwent SLNB in the present study.

Reasons for non-performance of SLNB included melanoma thickness below guideline thresholds, advanced patient age or relevant comorbidities, limited eligibility for subsequent systemic therapy, patient preference or refusal after informed discussion, prior excision at an external institution, or a multidisciplinary decision that SLNB was unlikely to influence clinical management. In such cases, patients were managed according to guideline-based surveillance strategies.

### 2.3. Sentinel Lymph Node Biopsy Procedure

Following histological verification of the primary melanoma, wide local excision was performed with margins determined according to contemporaneous NCCN guidelines. Preoperative lymphoscintigraphy was carried out in all patients to identify sentinel lymphatic drainage basins.

Sentinel lymph node biopsy was performed using a combined radiotracer and blue dye technique according to standard protocols. Preoperative dynamic lymphoscintigraphy was carried out on the day before surgery following peritumoral injection of technetium-99 m–labelled human albumin colloid (Sentiscint; Medi-Radiopharma Ltd., Érd, Hungary). As part of the dual-mapping approach, 0.5–2.0 mL of vital blue dye was injected intradermally 15–20 min prior to skin incision.

Intraoperatively, sentinel lymph nodes were identified using a handheld gamma probe (C-Trak, AEA Technology, Warrington, UK) and Navigator GPS System (RMD Instruments Corp. Watertown, MA, USA) in conjunction with visual identification of blue-stained lymphatic channels and nodes. Lymph nodes demonstrating significant radiotracer uptake and/or blue dye (Byk-Gulden, Konstanz, Germany) staining were considered sentinel lymph nodes and were excised. Ex vivo radioactivity counts were measured and compared with background activity in the surgical bed. Sentinel lymph node excision was continued until residual background counts were less than 10% of the activity of the hottest node removed.

### 2.4. Histopathological Examination

All excised skin specimens and sentinel lymph nodes were fixed in 4% buffered formaldehyde for 24 h at room temperature using a 1:10 tissue-to-fixative volume ratio. Representative sections of the primary tumor were submitted for routine histological evaluation, while sentinel lymph nodes were entirely embedded in paraffin blocks.

Serial 4-µm sections were cut and stained with hematoxylin–eosin (Leica ST5020; Leica Biosystems, Wetzlar, Germany). To increase diagnostic sensitivity, additional immunohistochemical analyses were performed using Melan-A (clone A103; Dako, Glostrup, Denmark) and/or HMB-45 (Biocare Medical, Pacheco, CA, USA) antibodies to detect isolated tumor cells and micrometastases. Automated immunohistochemical staining was performed using a Leica BOND-MAX (Leica Biosystems, Wetzlar, Germany) autostainer with a polymer-based horseradish peroxidase detection system, and all slides were counterstained with hematoxylin.

### 2.5. Postoperative Management

Patients with histopathologically positive SLNB findings underwent completion lymph node dissection and received intermediate-dose adjuvant interferon therapy (3 × 5–10 MIU IFN-2b subcutaneously). Patients with negative SLNB results and a Breslow thickness >1.5 mm received low-dose interferon therapy (3 × 3 MIU IFN-2a weekly for one year), in accordance with international guidelines in effect during the study period.

### 2.6. Endpoints and Outcome Definitions

The primary endpoints of the study were sentinel lymph node identification rate, false-omission rate (FOR), recurrence-free survival (RFS), and overall survival (OS).

For all time-to-event analyses, the date of primary tumor excision was used as the uniform index date (time zero), as this date was consistently available for all patients and represents a clinically relevant starting point for follow-up.

Overall survival was defined as the time from the date of primary tumor excision to death from any cause. Patients who were alive at last contact were censored at the date of last follow-up. Recurrence-free survival was defined as the time from the date of primary tumor excision to the first documented recurrence (local, satellite/in-transit, regional nodal, or distant metastasis). Patients without recurrence were censored at the date of last follow-up.

Regional nodal recurrence was defined as the occurrence of metastatic disease within the previously mapped and biopsied lymphatic basin following a negative SLNB, without preceding or simultaneous local or in-transit recurrence. Such events were classified as false-negative (FN) outcomes, consistent with definitions used in large head and neck melanoma SLNB series [24]. The false-omission rate was calculated as FN/(FN + TN). Patients without documented recurrence or death were censored at the date of last clinical follow-up.

### 2.7. Statistical Analysis

Survival outcomes were estimated using the Kaplan–Meier method. Associations between SLNB status and overall survival were evaluated using univariate Cox proportional hazards regression models, with negative SLNB serving as the reference category. Given the modest cohort size and limited number of events, multivariable analyses were not performed to avoid model overfitting.

Statistical analyses were conducted using SPSS software (version 26; IBM, Armonk, NY, USA). A *p*-value < 0.05 was considered statistically significant.

### 2.8. Literature Comparison

To contextualize the present findings, a structured review of previously published sentinel lymph node biopsy (SLNB) series in cutaneous head and neck melanoma was performed. Literature searches were conducted using PubMed and additional biomedical databases, applying combinations of the following keywords: “head and neck,” “melanoma,” “sentinel lymph node biopsy,” and “SLNB.” Titles and abstracts were screened to identify potentially relevant studies.

Full texts of potentially eligible articles were reviewed, and studies were included if they met the following predefined criteria: (1) inclusion of patients with primary cutaneous head and neck melanoma; (2) performance of sentinel lymph node biopsy; (3) inclusion of at least 75 patients undergoing SLNB; (4) reporting of follow-up duration; and (5) availability of nodal outcome metrics, including false-negative outcomes, false-omission rates, or sufficient data to derive these measures. Studies focusing solely on technical feasibility or mixed anatomical sites without head and neck–specific outcome data were excluded.

From the eligible studies, data on sentinel lymph node identification rate, reported nodal outcome metrics, and follow-up duration were extracted and summarized descriptively. Given heterogeneity in reporting standards across published series, the false-omission rate was selected as the primary comparative metric. No formal meta-analysis was performed.

## 3. Results

### 3.1. Patient and Tumor Characteristics

Thirty-eight patients with cutaneous melanoma of the head and neck underwent SLNB at the Department of Oral and Maxillofacial Surgery, University of Szeged. The median patient age was 52 years (range 21.3–77.9), with a median follow-up time of 6.8 years. Median Breslow thickness was 3.12 mm (range 0.5–18.0).

Twenty-three patients (60.5%) were male and 15 (39.5%) were female. Tumors were most frequently located on the scalp (47.4%), followed by the face (31.6%), ear (15.8%), and neck (5.2%). Pathological staging revealed that 25 patients (65.8%) had tumors classified as pT3a or lower, while 12 patients (31.6%) had pT3b or higher tumors. Detailed demographic and clinicopathological characteristics are summarized in Table 1.

### 3.2. SLNB Performance, Nodal Outcomes, and Survival

At least one sentinel lymph node (range 1–2) was successfully identified in all patients, yielding a sentinel lymph node identification rate of 100%. No major perioperative complications were observed. Minor wound infection occurred in two patients and resolved with conservative management.

Histopathological examination identified positive sentinel lymph nodes in eight patients (21.1%), while 30 patients (78.9%) had negative SLNB results. During follow-up, regional nodal recurrence occurred in two of the 30 patients with negative SLNB findings (6.6%). These events were classified as false-negative outcomes. Based on these findings, the false-omission rate was 6.7%, and the negative predictive value was 93.3%.

**Figure 1:**
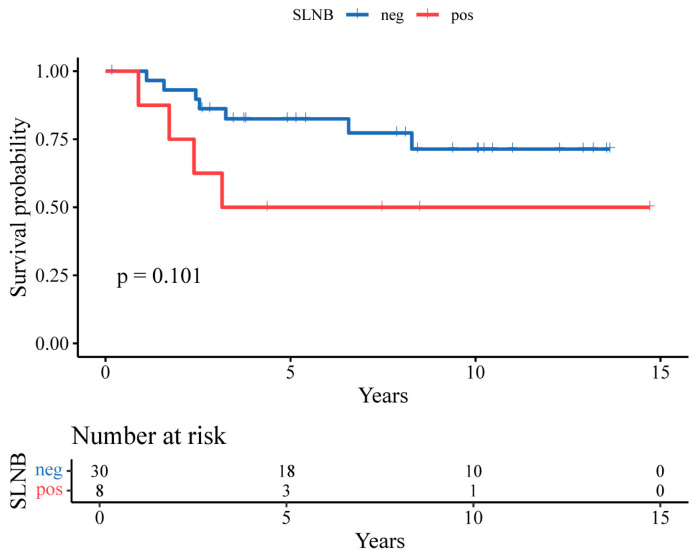
Kaplan–Meier overall survival (OS) curves for SLN-negative vs. SLN-positive patients.

**Figure 2:**
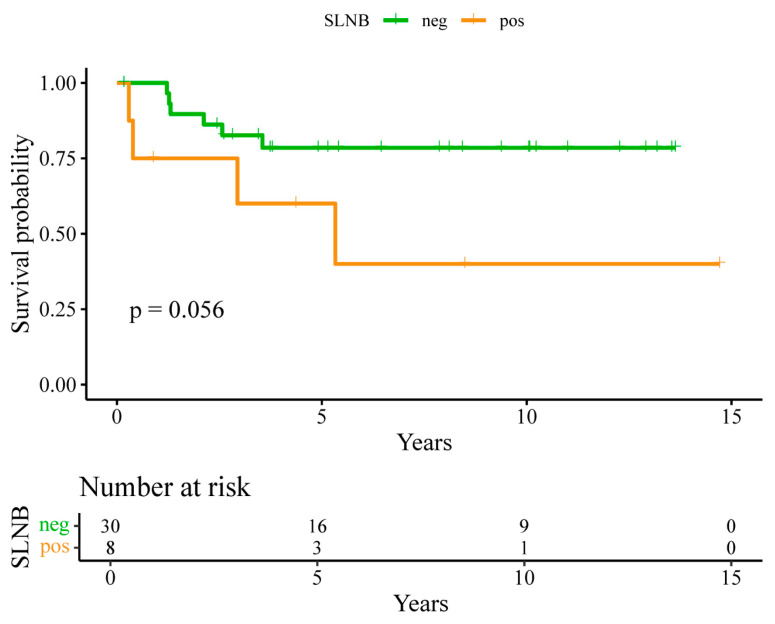
Kaplan–Meier recurrence-free survival (RFS) curves for SLN-negative vs. SLN-positive patients.

Kaplan–Meier survival analysis demonstrated longer RFS and OS among patients with negative SLNB findings compared with those with positive SLNB findings; however, these differences did not reach statistical significance (Figure 1 and Figure 2). In univariate Cox regression analyses, SLN positivity was associated with numerically higher hazards for recurrence and death; however, these associations were not statistically significant, and confidence intervals were wide, reflecting the limited number of events.

### 3.3. Intermediate-Risk Subgroup Analysis

Given that SLN histology remains a decisive factor in determining eligibility for adjuvant therapy, we conducted a separate analysis of patients with pT1b–pT3a melanoma—a subgroup for whom adjuvant treatment is not indicated under current guidelines without SLNB findings. This intermediate-risk subgroup included 27 patients, of whom five (18.5%) had positive SLNs, highlighting the clinical importance of SLNB in detecting patients who may benefit from closer surveillance or adjuvant therapy.

### 3.4. Comparison with Published Series

To contextualize our findings, sentinel lymph node identification rates, false-omission rates, and follow-up durations were compared with those reported in previously published large head and neck melanoma series (Table 2). In the present cohort, the 100% identification rate and the false-omission rate of 6.7% were within the ranges reported in these studies.

## 4. Discussion

In this study, we evaluated the clinical characteristics, nodal outcomes, and prognostic relevance of SLNB in patients with cutaneous head and neck melanoma treated at a medium-volume regional center. Although the number of patients undergoing SLNB was limited, the extended median follow-up of 6.8 years represents a key strength of this analysis, allowing reliable detection of delayed regional nodal recurrences and a meaningful assessment of false-omission rates in this anatomically complex disease.

It is well documented in the literature that SLNB is performed less frequently in patients with head and neck melanoma compared with those with melanomas arising at other anatomical sites. This observation does not necessarily indicate underutilization, but rather reflects the unique clinical, anatomical, and demographic characteristics of head and neck melanoma. Patients in this subgroup are often older, frequently present with lentigo maligna or lentigo maligna melanoma, and commonly have tumors that fall below guideline thresholds for SLNB. In addition, comorbidities and the anticipated impact of SLNB results on subsequent management play an important role in clinical decision-making.

Historically, adoption of SLNB in the head and neck region lagged behind that of other anatomical sites due to complex and variable lymphatic drainage patterns, the presence of bilateral nodal basins, and early reports of lower identification rates and higher false-negative rates. Early studies suggested a substantial learning curve before acceptable performance could be achieved. However, more recent evidence from specialized centers has demonstrated that, when key prerequisites are met—including multidisciplinary decision-making, dedicated head and neck surgical expertise, high-quality nuclear medicine imaging, and standardized histopathological evaluation—SLNB can be performed safely and effectively in the head and neck region, with diagnostic performance comparable to that reported from large published series.

Technological and diagnostic advances have further improved the reliability of SLNB in head and neck melanoma. The introduction of SPECT/CT has enhanced preoperative localization of sentinel lymph nodes in anatomically complex regions, reducing the likelihood of missed drainage pathways [28,29]. In parallel, refinements in histopathological processing, including serial sectioning and immunohistochemistry, have increased sensitivity for detecting micrometastatic disease [30]. Together, these developments have contributed to improved diagnostic accuracy and acceptable false-omission rates across institutions [31].

The clinical role of SLNB has evolved substantially in the contemporary melanoma treatment era. Following the results of the MSLT-II and DeCOG-SLT trials, completion lymph node dissection is no longer routinely recommended after a positive SLNB, underscoring that the principal value of SLNB lies primarily in accurate staging rather than routine regional disease control [32,33,34]. In this context, SLNB has become a pivotal step in risk stratification and selection of patients for adjuvant systemic therapy. In the present cohort, nearly one-fifth of patients in the intermediate-risk subgroup (pT1b–pT3a) had positive sentinel lymph nodes, highlighting the clinical importance of SLNB in identifying patients who may benefit from closer surveillance or consideration for adjuvant treatment. Beyond its established staging role, SLNB may also confer a limited therapeutic benefit in selected patients by enabling early removal of micrometastatic nodal disease [32].

When contextualized against previously published large head and neck melanoma series, the false-omission rate observed in our cohort (6.7%) falls within the range reported in the literature. As summarized in Table 2, reported false-omission rates vary across studies and appear to be influenced by patient selection, follow-up duration, and reporting practices. Notably, longer follow-up is often associated with higher observed false-omission rates, underscoring the importance of extended surveillance rather than inferior SLNB performance. Interpretation of these data is further complicated by heterogeneity in definitions and incomplete reporting of nodal outcome metrics across the literature, underscoring the need for standardized reporting in future studies.

While centralization of melanoma care remains an important principle, it is not universally feasible in all healthcare systems. In countries with smaller populations and geographically distributed care networks, referral of all patients with head and neck melanoma to a limited number of high-volume centers may be impractical and may delay timely treatment. In this context, it is essential that regional centers provide guideline-based, evidence-driven management rather than omitting recommended staging procedures solely due to lower institutional case volumes. Our findings support that, when appropriate infrastructure, multidisciplinary collaboration, and surgical expertise are available, SLNB can be delivered reliably in medium-volume centers, thereby ensuring equitable access to modern melanoma care.

This study has several limitations. Its retrospective design and relatively small cohort size limit statistical power and preclude multivariable analyses. Furthermore, the study was not designed to assess SLNB utilization rates among all head and neck melanoma patients, but rather to evaluate long-term nodal outcomes in patients who underwent SLNB within a standardized care pathway. Nevertheless, transparent reporting of patient selection, outcome definitions, and extended follow-up enables reliable interpretation of SLNB performance, providing a realistic benchmark for regional institutions.

Future research should focus on multicenter collaborations with standardized definitions of false-negative and false-omission rates, prospective capture of denominator data, and integration of advanced imaging and molecular risk stratification tools. Such efforts may further refine patient selection and optimize the role of SLNB within increasingly personalized melanoma care pathways.

## 5. Conclusions

Sentinel lymph node biopsy is a safe, reliable, and clinically meaningful staging procedure for patients with head and neck cutaneous melanoma. In this medium-volume regional center, SLNB achieved a 100% identification rate and a false-omission rate comparable to those reported by large published series, even after extended follow-up.

Sentinel node status provided important prognostic information and identified a clinically relevant proportion of intermediate-risk patients who would not otherwise be eligible for adjuvant systemic therapy based on primary tumor characteristics alone.

These findings indicate that, when performed within a multidisciplinary framework with appropriate surgical expertise, nuclear medicine support, and standardized histopathology, guideline-based SLNB can be delivered reliably outside high-volume tertiary centers.

## Figures and Tables

**Table 1:** Summary of demographic and tumor characteristics of the study population, including age, gender distribution, SLNB results, pathological stage, anatomical tumor site, Breslow thickness, and follow-up duration.

Characteristics		No. of Patients (%)
Mean age, years (range)		52.17 (21.3–77.9)
Median Follow-up, years		6.8
Gender	Male	23 (60.5)
	Female	15 (39.5)
SLNB	Negative	30 (78.9)
	Positive	8 (21.1)
Pathological stage	pT3a or lower	25 (65.8)
	pT3b or higher	12 (31.6)
	NA	1 (2.6)
Anatomical distribution	Scalp	18 (47.4)
	Face	12 (31.6)
	Ear	6 (15.8)
	Neck	2 (5.2)
Mean Breslow, mm (range)		3.12 (0.5–18.0)

**Table 2:** Comparative sentinel lymph node biopsy (SLNB) outcomes in previously published large head and neck melanoma series and the present study, including sentinel lymph node identification rates, false-omission rates (FOR), and reported follow-up durations.

Study/Center (Year)	No. of H&N Melanoma Cases (SLNB)	SLN Identification Rate (%)	FN-Related Metric Reported	False-Omission Rate (FOR, %)	Median/Mean Follow-Up
Carlson et al. (2005) [25]	125	98–100	FN, FOR	6.8	Mean 34.7 months
Miller et al. (2011) [19]	153	~99	FN, FNR, FOR	6.7	Median 28.8 months
Erman et al. (2012) [21]	353	99.7	FOR	4.2	~35 months (~2.9 yrs)
Hanks et al. (2020/2021) [24]	356	~99	FOR	6.4	Median 4.9 yrs
Evrard et al. (2018) [26]	124	97.6	FOR	7.1	Mean 46.6 months
Passmore-Webb et al. (2019) [27]	143	100	FN, FOR	2.6	Median ~33 months
Present study (Szeged, Hungary)	38	100	FN, FOR	6.7	Median 6.8 yrs

## Data Availability

Data are not publicly available due to privacy and ethical restrictions.

## Abbreviations

The following abbreviations are used in this manuscript:

AJCC	American Joint Committee on Cancer
CLND	Completion lymph node dissection
FOR	False-omission rate
FNR	False-negative rate
H&N	Head and neck
H&E	Hematoxylin and eosin
IHC	Immunohistochemistry
MSLT	Multicenter Selective Lymphadenectomy Trial
NCCN	National Comprehensive Cancer Network
NPV	Negative predictive value
OS	Overall survival
RFS	Recurrence-free survival
SLN	Sentinel lymph node
SLNB	Sentinel lymph node biopsy
SPECT/CT	Single-photon emission computed tomography/computed tomography
